# The association of systemic immune-inflammation index with incident breast cancer and all-cause mortality: evidence from a large population-based study

**DOI:** 10.3389/fimmu.2025.1528690

**Published:** 2025-01-24

**Authors:** Yu Li, Meng Yu, Ming Yang, Jingqi Yang

**Affiliations:** ^1^ Breast Surgery, Pingxiang People’s Hospital, Pingxiang, China; ^2^ Breast Surgery, Luxi County People’s Hospital, Pingxiang, China; ^3^ Department of Cardiovascular Medicine, Jiangxi Provincial People’s Hospital, The First Affiliated Hospital of Nanchang Medical College, Nanchang, China

**Keywords:** systemic immune-inflammation index, breast cancer, all-cause mortality, nonlinear relationship, nhaens

## Abstract

**Background:**

Chronic low-grade inflammation is recognized as a significant factor in various health outcomes, including the development and progression of breast cancer. The Systemic Immune-Inflammation Index (SII), a novel marker derived from routine blood counts, has been suggested as a predictor of all-cause mortality and cardiovascular mortality. However, its predictive value in a nationwide representative population, particularly for breast cancer incidence and mortality, is not well-established.

**Methods:**

This study aimed to assess the association of SII and the risk of breast cancer incidence and all-cause mortality in breast cancer patients within the National Health and Nutrition Examination Survey (NHANES) from 1999 to 2018. SII was calculated from complete blood count parameters. We used multifactor regression models to examine the associations between SII and the outcomes of interest.

**Results:**

A total of 21,058 female participants were included in the study, of which 557 (2.7%) were identified as having breast cancer. After adjusting for multiple potential confounders, the relationship between SII and the incidence of breast cancer revealed an inverse L-shaped association. The optimal inflection point for SII/100 was determined to be 5.09. Below this threshold, there was a significant increase in the risk of breast cancer (OR=1.05, 95% CI: 1.02-1.09). Within the breast cancer population, SII exhibited a J-shaped relationship with all-cause mortality. The optimal inflection point for SII/100 in this context was 5.22, and above this threshold, there was a marked escalation in all-cause mortality (HR=1.09, 95% CI: 1.04-1.14).

**Conclusion:**

The SII, as a novel inflammatory composite index, is significantly associated with the risk of breast cancer incidence and all-cause mortality in breast cancer patients. These findings highlight the importance of monitoring systemic inflammation and suggest that SII could serve as a valuable prognostic tool.

## Introduction

Breast cancer, a leading cause of cancer-related mortality among women worldwide, poses significant public health challenges with its incidence and mortality rates (1). This malignancy is characterized by a complex interplay of genetic, hormonal, and environmental factors that contribute to its development and progression (2, 3). The ability to predict incidence and prognosis at an early stage could lead to more precise treatment strategies and ultimately improve overall outcomes. Among the various factors implicated in breast cancer, inflammation has emerged as a key player in tumorigenesis (4, 5).

In the realm of cancer research, the role of immuno-inflammatory cells in tumorigenesis and progression is increasingly recognized (6, 7). The systemic immune-inflammation index (SII), a novel inflammatory marker, has been associated with poor prognosis in various cancers, including breast cancer (8–10). The SII, derived from the peripheral blood count of neutrophils, lymphocytes, and platelets, provides a comprehensive assessment of the inflammatory and immune status (11). It has been shown to correlate with tumor stage, grade, and response to treatment in breast cancer patients (12–14). The index offers a non-invasive and cost-effective means of reflecting the tumor’s microenvironmental immune-inflammatory state (15).

Recent studies have suggested that elevated SII values are associated with higher tumor stage, triple-negative breast cancer, and poorer responses to chemotherapy, indicating a significant correlation between SII and breast cancer clinicopathological characteristics (16, 17). Moreover, research has demonstrated that a high SII may predict poor survival in patients with breast cancer, particularly in those with hormone receptor-negative and HER2-positive disease (18). Despite the growing interest in SII, there is a paucity of literature focusing on its relationship with breast cancer incidence and the prediction of long-term mortality rates remains an enigma.

In this study, we aim to investigate the relationship between SII and the risk of breast cancer, as well as its association with mortality rates among women diagnosed with breast cancer. By leveraging the extensive data from the National Health and Nutrition Examination Survey (NHANES) from 1999 to 2018, we hope to contribute to the understanding of the role of systemic inflammation in breast cancer and identify potential prognostic factors that could inform clinical practice and public health strategies aimed at mitigating the impact of this disease.

## Methods

### Data selection and study design

**Figure 1:**
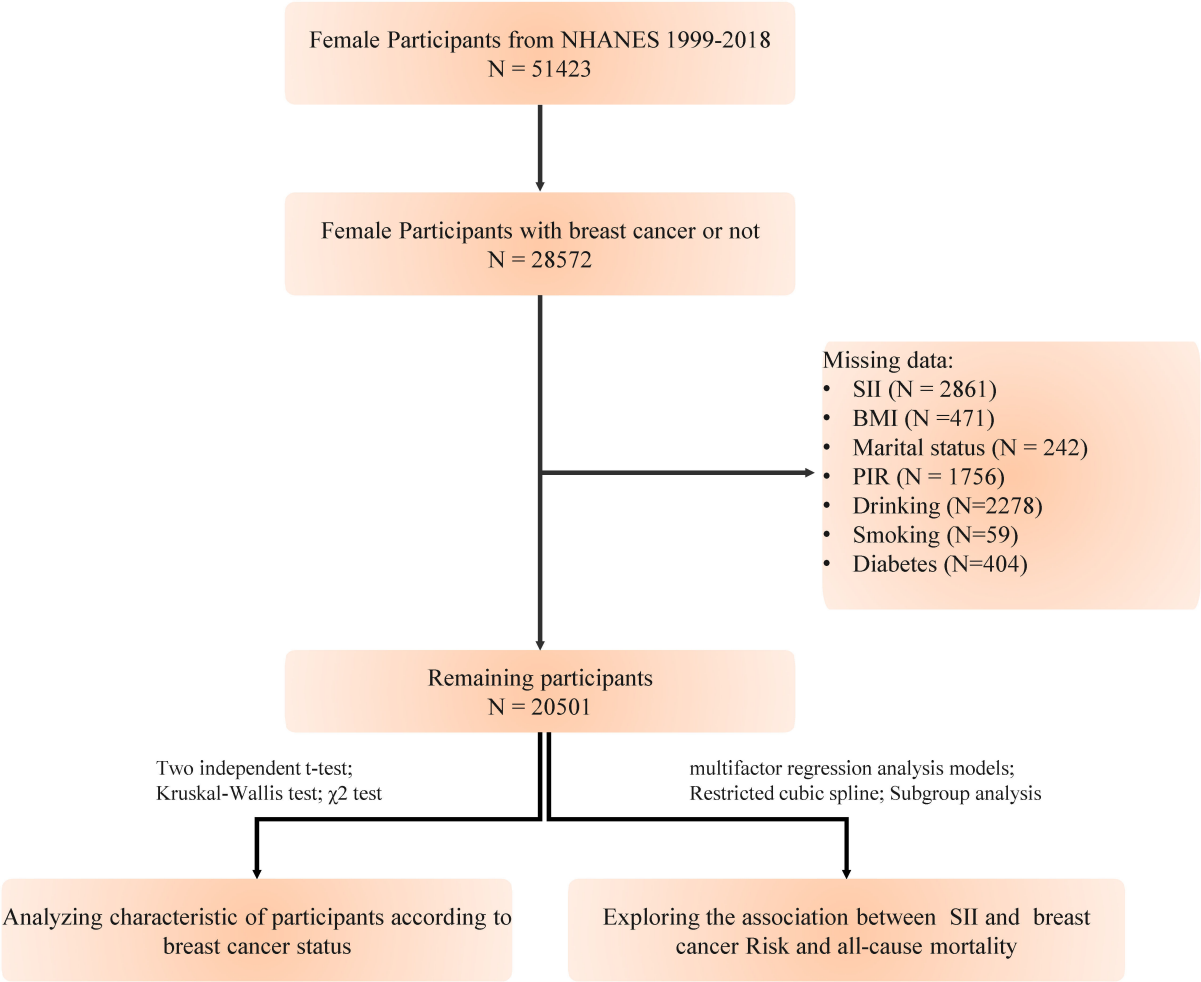
Study flowchart.

We extracted data from the National Health and Nutrition Examination Survey (NHANES, https://www.cdc.gov/nchs/nhanes/index.htm) database, which annually surveys a representative sample of approximately 5,000 individuals nationwide. The database encompasses a comprehensive range of information, including demographics, dietary habits, examination results, laboratory measurements, questionnaire responses, and limited access data. NHANES has been conducted over ten cycles from 1999 to 2018. This study was approved by the National Center for Health Statistics Research Ethics Review Board, and all participants provided written informed consent. Our study excluded the following (1): individuals under 18 years of age (2); pregnant women (3); participants with a history of other types of cancer (4); those with missing data on breast cancer diagnosis (5); individuals lacking data on platelet, neutrophil, and lymphocyte counts. After applying these exclusions, a total of 20,501 participants were included in the study (**Figure 1**). The objective of this investigation is to explore the correlation between the SII and the risk of developing breast cancer, as well as its link to all-cause mortality in women with breast cancer.

### Definition of SII and breast cancer

Complete blood count (CBC) parameters were derived using the Beckman Coulter methodology, which involves counting and sizing blood cells with an automatic dilution and mixing device, followed by hemoglobinometry using a single-beam photometer. The white blood cell differential was assessed using VCS technology. The Beckman Coulter DxH 800 instrument, located in the NHANES mobile examination center (MEC), generates a CBC for each participant, providing a detailed distribution of blood cells. The SII was calculated using the formula: platelet count × neutrophil count/lymphocyte count. To facilitate analysis, SII values were standardized by dividing by 100, accounting for the typically high values of these inflammatory markers.

Breast cancer cases were identified through participant responses to the question, “What kind of cancer do you have?” which allowed us to categorize individuals into breast cancer and non-breast cancer groups. All relevant data are accessible on the website of the Centers for Disease Control and Prevention.

### Assessment of mortality

Mortality data for our study were sourced from the National Death Index (NDI) death certificate records, as provided by the National Center for Health Statistics (NCHS), with updates through December 31, 2019. The study’s endpoints encompassed all-cause mortality. Causes of death were classified according to the International Statistical Classification of Diseases, 10th Revision (ICD-10). All-cause mortality was defined as death from any cause, including but not limited to heart disease (ICD-10 codes 054–068), malignant neoplasms (ICD-10 codes 019–043), accidents (unintentional injuries, ICD-10 codes 112–123), cerebrovascular diseases (ICD-10 code 070), diabetes mellitus (ICD-10 code 046), and other specified causes. Follow-up duration was calculated from the baseline interview date until the date of death or December 31, 2019, whichever came first.

### Covariates

Age was determined by calculating the difference between the interview date and the participant’s date of birth. Race and ethnicity were assessed using two questions (1): “Do you consider yourself Hispanic, Latino, or Spanish?” and (2) “What is your race?” Responses to these questions allowed NHANES staff to categorize participants into five racial/ethnic groups: Mexican American, Other Hispanic, Non-Hispanic White, Non-Hispanic Black, and Other Race. Education level was categorized based on the question, “What is the highest grade or level of school you have completed or the highest degree you have received?” which resulted in three groups: less than high school, high school graduate, and some college or associate degree or higher. Marital status was classified as married, never married, or other. The poverty income ratio (PIR) is the ratio of the family’s self-reported income to the poverty threshold for their family size. Body mass index (BMI) was calculated as weight in kilograms divided by the square of height in meters (kg/m^2). Two BMI categories were used: <25 and ≥25 kg/m^2, indicating underweight/normal weight and overweight/obesity, respectively. Smoking and alcohol consumption were dichotomized into yes or no categories. Diabetes was identified based on a documented medical history in the participants’ records.

### Statistical analysis

The significance level for all tests was set at P<0.05. All analyses were conducted using R version 3.4.3 and Empower(R) version 4.2 software. Descriptive statistics were used to summarize the data, with means and standard deviations (SDs) presented for continuous variables with a normal distribution, medians and interquartile ranges (IQRs) for non-normally distributed continuous variables, and proportions for categorical variables. Differences in continuous variables between groups were assessed using Student’s t-test, while categorical variables were compared using the χ2 test. Prior to modeling, variance inflation factors were calculated to detect potential multicollinearity (19). A multivariate logistic regression model was employed to determine the odds ratios (ORs) and 95% confidence intervals (95% CIs) for the association between SII and breast cancer. Similarly, a multivariate Cox regression model was utilized to estimate the relationship between SII and all-cause mortality among breast cancer patients. Three models were adjusted for covariates as follows: Model 1 was unadjusted; Model 2 was adjusted for age and race; Model 3 included the adjustments of Model 2 plus PIR, education, marital status, BMI, smoking, alcohol consumption, and diabetes. Kaplan-Meier (KM) survival curves were constructed to plot the survival probabilities over time, comparing breast cancer patients with non-breast cancer individuals, based on the duration from the baseline interview to the date of death or the end of follow-up. The log-rank test was employed to statistically evaluate the differences in survival distributions between the two groups. Additionally, we incorporated restricted cubic splines (RCS) with four knots in both logistic and Cox regression models to model the dose-response relationship between SII and the outcomes. When nonlinear relationships were detected, recursive algorithms were used to identify potential inflection points, and segmented regressions were applied to assess the hazard ratios (HRs) associated with SII before and after these points. Stratified analyses were conducted based on SII, stratified by age (less than 60 or 60 years or older), BMI (less than 25 or 25 kg/m^2 or greater), alcohol consumption (yes or no), smoking status (yes or no), and diabetes (yes or no). Multiplicative interaction terms were included to assess whether the relationship between SII and breast cancer risk was modified by the aforementioned factors.

## Results

### Participant characteristics

A total of 20,501 female participants were included in the study, of which 557 (2.7%) were identified as having breast cancer. **Table 1** summarizes the baseline characteristics of the participants categorized by breast cancer status. The group with breast cancer was older, had a higher proportion of Non-Hispanic White individuals, fewer never-married individuals, a higher PIR, a greater prevalence of smoking, a lower prevalence of alcohol consumption, and a lower prevalence of diabetes.

**Table 1:** Baseline characteristics of the participants with and without breast cancer.

	Non-Breast cancer (N=19944)	Breast cancer (N=557)	P-value
Age (Years)	47.00 (33.00-63.00)	69.00 (60.00-77.00)	<0.01
BMI (kg/m2)	28.20 (24.01-33.44)	27.61 (24.14-32.50)	0.07
Race (%)			<0.01
Mexican American	3495 (17.52%)	48 (8.62%)	
Other Hispanic	1664 (8.34%)	27 (4.85%)	
Non-Hispanic White	9116 (45.71%)	362 (64.99%)	
Non-Hispanic Black	3976 (19.94%)	85 (15.26%)	
Others	1693 (8.49%)	35 (6.28%)	
Marital status (%)			<0.01
Married	9758 (48.93%)	263 (47.22%)	
Never married	3273 (16.41%)	29 (5.21%)	
Others	6913 (34.66%)	265 (47.58%)	
Education (%)			0.29
Less than high school	4826 (24.20%)	122 (21.90%)	
High school or equivalent	4469 (22.41%)	123 (22.08%)	
College or above	10649 (53.39%)	312 (56.01%)	
PIR (%)			<0.01
<1	4240 (21.26%)	83 (14.90%)	
≥1, <3	8395 (42.09%)	239 (42.91%)	
≥3	7309 (36.65%)	235 (42.19%)	
Smoking status (%)			<0.01
Never	12568 (63.02%)	324 (58.17%)	
Now	3531 (17.70%)	61 (10.95%)	
Former	3845 (19.28%)	172 (30.88%)	
Drinking (%)			<0.01
Never	7475 (37.48%)	244 (43.81%)	
Every day or nearly every day	3456 (17.33%)	93 (16.70%)	
3 to 4 times a week	2852 (14.30%)	56 (10.05%)	
1 to 2 times a week	4431 (22.22%)	116 (20.83%)	
Less than once a week	1730 (8.67%)	48 (8.62%)	
Diabetes (%)			<0.01
Yes	17364 (87.06%)	432 (77.56%)	
No	2580 (12.94%)	125 (22.44%)	

Data were presented as median (Interquartile range) or n (%).

TyG, Triglyceride-glucose index; BMI, Body Mass Index; PIR, Poverty Impact Ratio.

### Association of the SII with breast cancer risk and all-cause mortality

Prior to constructing the multivariable logistic regression analysis, we examined the collinearity between the SII and other covariates. The results indicated that the VIF for all covariates included in this study was less than 5, suggesting that there is no collinearity between the SII and the other covariates (**Supplementary Table 1**).

After confirming the absence of collinearity, we proceeded to analyze the associations between SII and breast cancer outcomes. **Table 2** presents the associations between the SII and the risk of breast cancer, as well as all-cause mortality among breast cancer patients. In the fully adjusted model, we observed a significant association between elevated SII levels and an increased risk of breast cancer. Specifically, for every 100-unit increase in SII, the odds of breast cancer were significantly higher (OR = 1.02, 95% CI: 1.00, 1.04). When SII was categorized into quartiles, the highest quartile showed a robust association with breast cancer risk compared to the lowest quartile, with an adjusted odds ratio of 1.46 (95% CI: 1.14, 1.86, P < 0.01).

**Table 2:** Association of the SII with breast cancer risk and all-cause mortality.

Exposure	Non-adjusted	Adjust I	Adjust II
Breast cancer			
SII/100	1.02 (1.00, 1.04) 0.02	1.02 (1.00, 1.04) 0.03	1.02 (1.00, 1.04) 0.02
Q1	1.0	1.0	1.0
Q2	1.08 (0.85, 1.38) 0.52	1.13 (0.88, 1.45) 0.33	1.13 (0.88, 1.45) 0.33
Q3	0.94 (0.73, 1.21) 0.65	1.01 (0.78, 1.30) 0.96	1.00 (0.78, 1.30) 0.97
Q4	1.32 (1.04, 1.66) 0.02	1.42 (1.12, 1.82) <0.01	1.46 (1.14, 1.86) <0.01
P for trend	0.04	0.01	<0.01
All-cause mortality in breast cancer			
SII	1.08 (1.04, 1.11) <0.01	1.06 (1.02, 1.10) <0.01	1.07 (1.03, 1.11) <0.01
Q1	1.0	1.0	1.0
Q2	1.11 (0.68, 1.80) 0.69	1.25 (0.77, 2.05) 0.37	1.14 (0.68, 1.90) 0.62
Q3	1.15 (0.71, 1.88) 0.57	0.91 (0.56, 1.50) 0.72	0.89 (0.53, 1.50) 0.66
Q4	1.73 (1.12, 2.68) 0.01	1.47 (1.08, 2.29) 0.03	1.41 (1.10, 2.27) 0.03
P for trend	0.04	0.01	<0.01

Non-adjusted model adjust for: None.

Adjust I model adjust for: Age; Race.

Adjust II model adjust for: Age; BMI; Race; Marital status; Education; PIR; Smoking status; Drinking; Diabetes.

The KM survival analysis showed a significantly lower survival rate in the breast cancer group compared to the non-breast cancer group during the follow-up period (P<0.01, **Supplementary Figure 1**). These findings highlight the substantial impact of breast cancer on survival outcomes. Therefore, in addition to examining the relationship between the SII and the risk of breast cancer, we also analyzed the relationship between SII and the prognosis of breast cancer patients, which has significant implications for their prognosis. In the multivariable Cox regression analysis, a clear association was observed between all-cause mortality and SII in breast cancer patients. The hazard ratio for all-cause mortality associated with a 100-unit increase in SII was 1.07 (95% CI: 1.03, 1.11, P < 0.01) in the fully adjusted model. This indicates that for every 100-unit increase in SII, there was a 7% increase in the risk of all-cause mortality. Similar to the risk of breast cancer, the highest quartile of SII was associated with a significantly increased risk of all-cause mortality compared to the lowest quartile, with a hazard ratio of 1.41 (95% CI: 1.10, 2.27, P = 0.03) in the fully adjusted model.

### Non-linear relationship of SII with breast cancer risk and all-cause mortality in breast cancer patients

**Figure 2:**
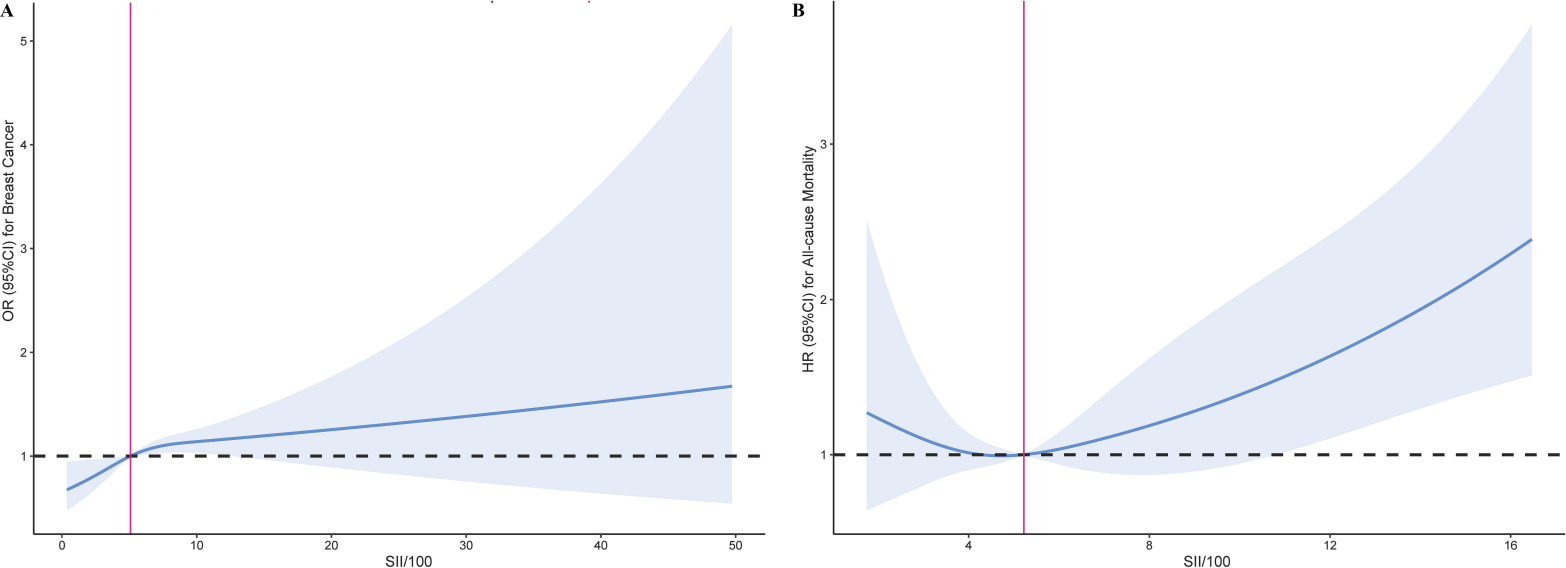
Restricted cubic spline curve for the association of SII with breast cancer risk and all-cause mortality. **(A)** Association between SII and the risk of breast cancer. **(B)** Association between SII and the all-cause mortality in breast cancer.

The non-linear associations between the SII and the risk of breast cancer, as well as all-cause mortality in breast cancer patients, were investigated using RCS models. The RCS analysis revealed an inverse L-shaped association between SII and the risk of breast cancer, with an inflection point at 5.09 for SII/100 (**Figure 2A**). Below this inflection point, there was a significant increase in the risk of breast cancer with an adjusted OR of 1.05 (95% CI: 1.02, 1.09, **Table 3**). Above the inflection point, there was no significant association, with an OR of 0.99 (95% CI: 0.94, 1.03, **Table 3**). In contrast, SII exhibited a J-shaped relationship with all-cause mortality in breast cancer patients, with the inflection point established at 5.22 for SII/100 (**Figure 2B**). SII values beneath this inflection point were not significantly linked to mortality, yielding an adjusted hazard ratio (HR) of 0.93 (95% CI: 0.78, 1.10, **Table 3**). However, for SII values surpassing the inflection point, there was an increase in all-cause mortality, with an HR of 1.09 (95% CI: 1.04, 1.14, **Table 3**). These findings underscore the complex, non-linear dynamics of SII in relation to breast cancer outcomes.

**Table 3:** Threshold effect analysis of SII on breast cancer risk and all-cause mortality.

	Breast Cancer	All-cause mortality
Inflection point	5.09	5.22
< Inflection point	1.05 (1.02, 1.09) <0.01	0.93 (0.78, 1.10) 0.39
> Inflection point	0.99 (0.94, 1.03) 0.53	1.09 (1.04, 1.14) <0.01
P for Log-likelihood ratio	0.03	0.02

Adjust for age, BMI, race, marital status, education, PIR, smoking status, drinking and diabetes.

### Subgroup analysis

**Figure 3:**
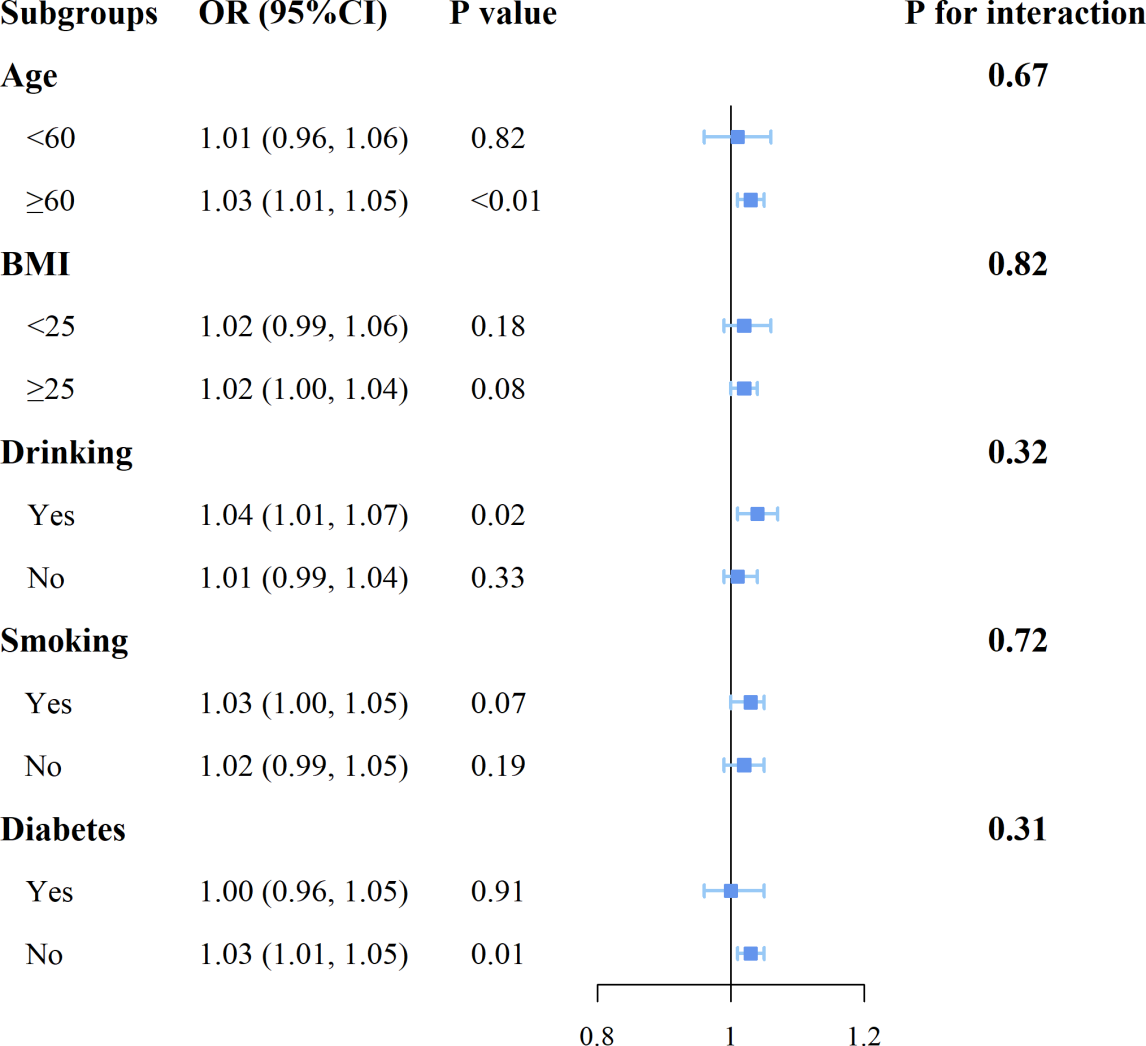
Forest plot for subgroup analysis of associations between SII and the risk of breast cancer.

As shown in **Figure 3**, the results of the subgroup analyses by age, BMI, drinking status, smoking status, and diabetes status indicate that in individuals aged 60 or older, drinkers, and those without diabetes, SII shows a more pronounced positive correlation with breast cancer (P<0.05). Additionally, no significant interactions were found between SII and these stratification variables.

## Discussion

In our large-scale study involving 20,501 female participants aged 18 years or older, we identified non-linear relationships between the SII and significant prognostic factors of breast cancer, including the risk of developing the disease and all-cause mortality among affected patients. The breast cancer risk exhibited an inverse L-shaped relationship, while all-cause mortality demonstrated a J-shaped relationship with SII. These associations were consistent across various subgroups, suggesting a clinical potential for SII in the prediction of breast cancer risk and prognosis.

The SII has been gaining attention as an inflammatory marker with prognostic capabilities across various diseases, including cancer (20–22). When compared with other inflammation indices, SII demonstrates a distinct advantage in its predictive power. SII has been found to be a stronger independent predictor compared to other markers such as the neutrophil-to-lymphocyte ratio (NLR) and platelet-to-lymphocyte ratio (PLR) (23). In a study investigating the correlation between SII and the degree of coronary artery stenosis in patients with coronary heart disease, SII emerged as the most potent independent predictor (24). This suggests that SII may be more sensitive in reflecting the inflammatory state associated with disease severity and prognosis. As a composite of neutrophil, lymphocyte, and platelet counts, SII provides an objective reflection of the body’s inflammatory state. Despite its established predictive power in multiple disease contexts, the application of SII in oncology, particularly in breast cancer prognosis, remains less explored compared to other inflammatory indices. This gap is notable as SII’s ability to reflect the balance between inflammation and immune function positions it as a potentially valuable biomarker in cancer management.

Our study, which may be the first to focus on the inverse L-shaped relationship between the SII and the risk of breast cancer, included adult participants from the NHANES database spanning from 1999 to 2018. This indicates that as SII increases, particularly below the established threshold, there is a significant elevation in the risk of breast cancer, suggesting the presence of a pro-inflammatory environment that may promote tumorigenesis and development. The potential mechanisms are likely multifactorial. SII, as an indicator of systemic inflammation, reflects the balance between pro-inflammatory neutrophils and anti-inflammatory lymphocytes, as well as platelet activity, which is known to be involved in cancer progression. Elevated neutrophils can contribute to the release of cytokines that stimulate angiogenesis and tumor growth, while a reduction in lymphocytes may signify an impaired immune surveillance, allowing for uncontrolled tumor expansion. Additionally, platelets not only play a role in clotting but also release factors that can enhance tumor cell adhesion, migration, and invasion. The observed relationship may thus be a result of these complex interactions within the tumor microenvironment, which are influenced by systemic inflammatory processes.

In the NHANES database, due to the limited number of breast cancer cases, which only numbered 557, we were able to analyze the prognosis of all-cause mortality. The results revealed a J-shaped relationship between SII and all-cause mortality, indicating that the risk of death escalates when SII surpasses this threshold. This suggests a potential limit to the immune system’s capacity to control disease progression at elevated SII levels. This is consistent with recent studies that have highlighted the prognostic significance of SII in various cancers (25–27).

The mechanisms underlying the association between the SII and all-cause mortality in breast cancer patients are not fully understood. However, emerging evidence points to several potential pathways. SII, which encompasses neutrophil, lymphocyte, and platelet counts, reflects the complex interplay between inflammation and immune response in cancer progression. Neutrophils not only alter the tumor microenvironment by promoting tumor cell proliferation, invasion, and metastasis through the extrinsic pathway but also secrete inflammatory mediators that can drive cellular senescence via the intrinsic pathway (28, 29). This dual role of neutrophils suggests that they may contribute to both the spread and the resistance of tumor cells to apoptosis, thereby influencing mortality in breast cancer patients (30). Platelets have been shown to activate and act as chemoattractants for cancer cells, creating favorable conditions for metastatic foci and promoting the epithelial-to-mesenchymal transition in tumor cells, which is crucial for metastasis (31, 32). Platelets can increase the level of circulating tumor cells, further facilitating the spread of cancer (33). Lymphopenia may aid tumor cells in evading immune surveillance and protect them from damage by cytotoxic T cells, thereby potentially promoting tumor progression and reducing survival (34). This suggests that the balance of immune cells, as reflected by SII, is critical in the immune response to breast cancer.

The present study boasts several significant advantages that contribute to its reliability and impact. Firstly, the study benefits from the large sample size and complex, multi-stage probability sampling design inherent in the NHANES database, which provides a robust platform for deriving reliable conclusions with strong statistical power. Secondly, by controlling for numerous known risk factors, we have effectively mitigated potential confounding variables in our analysis of the novel inflammatory index, SII, in relation to breast cancer risk. Lastly, this study is pioneering in revealing the non-linear relationship between SII and the incidence of breast cancer. Our findings offer novel insights that could significantly influence clinical practice and shape future research directions. The identification of an inverse L-shaped relationship between SII and breast cancer risk is a discovery that adds a new dimension to our understanding of systemic inflammation in cancer. Despite these strengths, our study is not without limitations. Firstly, the sample size of individuals with breast cancer within the NHANES database is relatively small, which may limit the generalizability of our results to other populations with different demographic characteristics. Secondly, the NHANES database lacks detailed clinical and pathological information for individual cancer cases, such as tumor stage, grade, and specific treatments received. This omission precludes a more nuanced analysis of how these factors may interact with SII levels and influence survival outcomes. Thirdly, the cross-sectional design of the NHANES data restricts our ability to establish causality between SII levels and breast cancer outcomes. Additionally, there is a potential for unmeasured confounders that could influence the relationship between SII and breast cancer outcomes. Although we adjusted for several potential confounders, other variables not captured in the NHANES database might affect our results. Lastly, the reliance on self-reported information and the completeness of follow-up in the NHANES database could introduce bias or incompleteness in our survival analysis, affecting the accuracy of our mortality data.

In conclusion, our study has identified significant non-linear relationships between the SII and breast cancer outcomes within the NHANES cohort. An inverse L-shaped relationship was observed between SII and the risk of breast cancer, while a J-shaped relationship was noted between SII and all-cause mortality among breast cancer patients, which indicates that both low and high levels of SII may be associated with risks related to breast cancer. These findings underscore the importance of understanding and addressing chronic inflammation in the context of breast cancer, potentially offering a promising strategy for risk reduction and improved prognosis in affected individuals. Further research is warranted to explore the therapeutic potential of modulating SII levels and to confirm these associations in diverse populations.

## Data Availability

Publicly available datasets were analyzed in this study. The data of NHANES can be downloaded from the website: https://wwwn.cdc.gov/nchs/nhanes/default.aspx. Further inquiries can be directed to the corresponding author.

## References

[1] Trapani D Ginsburg O Fadelu T Lin NU Hassett M Ilbawi AM. Global challenges and policy solutions in breast cancer control. Cancer Treat Rev. (2022) 104:102339. doi: 10.1016/j.ctrv.2022.102339 35074727

[2] Xu H Xu B. Breast cancer: epidemiology, risk factors and screening. Chin J Cancer Res. (2023) 35:565–83. doi: 10.21147/j.issn.1000-9604.2023.06.02 PMC10774137 38204449

[3] Coughlin SS. Epidemiology of breast cancer in women. Adv Exp Med Biol. (2019) 1152:9–29. doi: 10.1007/978-3-030-20301-6_2 31456177

[4] Bahiraee A Ebrahimi R Halabian R Aghabozorgi AS Amani J. The role of inflammation and its related micrornas in breast cancer: A narrative review. J Cell Physiol. (2019) 234:19480–93. doi: 10.1002/jcp.28742 31025369

[5] Göbel A Dell’Endice S Jaschke N Pählig S Shahid A Hofbauer LC. The role of inflammation in breast and prostate cancer metastasis to bone. Int J Mol Sci. (2021) 22:5078. doi: 10.3390/ijms22105078 34064859 PMC8151893

[6] Schmitt M Greten FR. The inflammatory pathogenesis of colorectal cancer. Nat Rev Immunol. (2021) 21:653–67. doi: 10.1038/s41577-021-00534-x 33911231

[7] Ozga AJ Chow MT Luster AD. Chemokines and the immune response to cancer. Immunity. (2021) 54:859–74. doi: 10.1016/j.immuni.2021.01.012 PMC8434759 33838745

[8] Zhu M Chen L Kong X Wang X Li X Fang Y. The systemic immune-inflammation index is an independent predictor of survival in breast cancer patients. Cancer Manag Res. (2022) 14:775–820. doi: 10.2147/cmar.S346406 35241935 PMC8887616

[9] Terasaki F Sugiura T Okamura Y Ito T Yamamoto Y Ashida R. Systemic immune-inflammation index as a prognostic marker for distal cholangiocarcinoma. Surg Today. (2021) 51:1602–9. doi: 10.1007/s00595-021-02312-7 34142236

[10] Xu S Cao S Yu Y. High systemic immune-inflammation index is a predictor of poor prognosis in patients with nonsmall cell lung cancer and bone metastasis. J Cancer Res Ther. (2021) 17:1636–42. doi: 10.4103/jcrt.jcrt_176_21 35381733

[11] Liu B Wang J Li YY Li KP Zhang Q. The association between systemic immune-inflammation index and rheumatoid arthritis: evidence from nhanes 1999-2018. Arthritis Res Ther. (2023) 25:34. doi: 10.1186/s13075-023-03018-6 36871051 PMC9985219

[12] Hong YM Yoon KT Cho M. Systemic immune-inflammation index predicts prognosis of sequential therapy with sorafenib and regorafenib in hepatocellular carcinoma. BMC Cancer. (2021) 21:569. doi: 10.1186/s12885-021-08124-9 34006248 PMC8130266

[13] Yuan X Feng H Huang H Li J Wu S Yuan Y. Systemic immune-inflammation index during treatment predicts prognosis and guides clinical treatment in patients with nasopharyngeal carcinoma. J Cancer Res Clin Oncol. (2023) 149:191–202. doi: 10.1007/s00432-022-04506-z 36595043 PMC9889477

[14] Zhang X Liu Q. Systemic immune inflammation index and T-staging predict prognosis in patients with muscle-invasive bladder cancer. Arch Esp Urol. (2023) 76:511–8. doi: 10.56434/j.arch.esp.urol.20237607.63 37867336

[15] Li C Wu J Jiang L Zhang L Huang J Tian Y. The predictive value of inflammatory biomarkers for major pathological response in non-small cell lung cancer patients receiving neoadjuvant chemoimmunotherapy and its association with the immune-related tumor microenvironment: A multi-center study. Cancer Immunol Immunother. (2023) 72:783–94. doi: 10.1007/s00262-022-03262-w PMC10991885 36056951

[16] Liu J Shi Z Bai Y Liu L Cheng K. Prognostic significance of systemic immune-inflammation index in triple-negative breast cancer. Cancer Manag Res. (2019) 11:4471–80. doi: 10.2147/cmar.S197623 PMC6526193 31191009

[17] Zhou Y Guo X Shen L Liu K Sun Q Wang Y. Predictive significance of systemic immune-inflammation index in patients with breast cancer: A retrospective cohort study. Onco Targets Ther. (2023) 16:939–60. doi: 10.2147/ott.S434193 PMC10658965 38021447

[18] Sun Y Li W Li AJ Su H Yue J Yu J. Increased systemic immune-inflammation index independently predicts poor survival for hormone receptor-negative, her2-positive breast cancer patients. Cancer Manag Res. (2019) 11:3153–62. doi: 10.2147/cmar.S190335 PMC6489660 31114357

[19] Kim JH. Multicollinearity and misleading statistical results. Korean J Anesthesiol. (2019) 72:558–69. doi: 10.4097/kja.19087 PMC6900425 31304696

[20] Li W Wang X Diao H Yang Y Ding L Huan W. Systemic immune inflammation index with all-cause and cause-specific mortality: A meta-analysis. Inflammation Res. (2024) 73:2199–216. doi: 10.1007/s00011-024-01959-5 39400697

[21] Yan X Huang J Chen X Lin M. Association between increased systemic immune-inflammation index and postoperative delirium in older intertrochanteric fracture patients. J Orthop Surg Res. (2024) 19:219. doi: 10.1186/s13018-024-04699-8 38566241 PMC10988850

[22] Yang C Yang Q Xie Z Peng X Liu H Xie C. Association of systemic immune-inflammation-index with all-cause and cause-specific mortality among type 2 diabetes: A cohort study base on population. Endocrine. (2024) 84:399–411. doi: 10.1007/s12020-023-03587-1 38048013 PMC11076376

[23] Elbeyli A Kurtul BE. Systemic immune-inflammation index, neutrophil-to-lymphocyte ratio, and platelet-to-lymphocyte ratio levels are associated with keratoconus. Indian J Ophthalmol. (2021) 69:1725–9. doi: 10.4103/ijo.IJO_3011_20 PMC8374788 34146015

[24] Liu Y Ye T Chen L Jin T Sheng Y Wu G. Systemic immune-inflammation index predicts the severity of coronary stenosis in patients with coronary heart disease. Coron Artery Dis. (2021) 32:715–20. doi: 10.1097/mca.0000000000001037 33826540

[25] Liu J Li S Zhang S Liu Y Ma L Zhu J. Systemic immune-inflammation index, neutrophil-to-lymphocyte ratio, platelet-to-lymphocyte ratio can predict clinical outcomes in patients with metastatic non-small-cell lung cancer treated with nivolumab. J Clin Lab Anal. (2019) 33:e22964. doi: 10.1002/jcla.22964 31282096 PMC6805305

[26] Shui Y Li M Su J Chen M Gu X Guo W. Prognostic and clinicopathological significance of systemic immune-inflammation index in pancreatic cancer: A meta-analysis of 2,365 patients. Aging (Albany NY). (2021) 13:20585–97. doi: 10.18632/aging.203449 PMC8436945 34435973

[27] Hu B Yang XR Xu Y Sun YF Sun C Guo W. Systemic immune-inflammation index predicts prognosis of patients after curative resection for hepatocellular carcinoma. Clin Cancer Res. (2014) 20:6212–22. doi: 10.1158/1078-0432.Ccr-14-0442 25271081

[28] Felix K Gaida MM. Neutrophil-derived proteases in the microenvironment of pancreatic cancer -active players in tumor progression. Int J Biol Sci. (2016) 12:302–13. doi: 10.7150/ijbs.14996 PMC4753159 26929737

[29] Moses K Brandau S. Human neutrophils: their role in cancer and relation to myeloid-derived suppressor cells. Semin Immunol. (2016) 28:187–96. doi: 10.1016/j.smim.2016.03.018 27067179

[30] Yu X Li C Wang Z Xu Y Shao S Shao F. Neutrophils in cancer: dual roles through intercellular interactions. Oncogene. (2024) 43:1163–77. doi: 10.1038/s41388-024-03004-5 38472320

[31] Orellana R Kato S Erices R Bravo ML Gonzalez P Oliva B. Platelets enhance tissue factor protein and metastasis initiating cell markers, and act as chemoattractants increasing the migration of ovarian cancer cells. BMC Cancer. (2015) 15:290. doi: 10.1186/s12885-015-1304-z 25886038 PMC4410584

[32] Labelle M Begum S Hynes RO. Direct signaling between platelets and cancer cells induces an epithelial-mesenchymal-like transition and promotes metastasis. Cancer Cell. (2011) 20:576–90. doi: 10.1016/j.ccr.2011.09.009 PMC3487108 22094253

[33] Kanikarla-Marie P Lam M Menter DG Kopetz S. Platelets, circulating tumor cells, and the circulome. Cancer Metastasis Rev. (2017) 36:235–48. doi: 10.1007/s10555-017-9681-1 28667367

[34] Ménétrier-Caux C Ray-Coquard I Blay JY Caux C. Lymphopenia in cancer patients and its effects on response to immunotherapy: an opportunity for combination with cytokines? J Immunother Cancer. (2019) 7:85. doi: 10.1186/s40425-019-0549-5 30922400 PMC6437964

