# Supplementary material for: The association of systemic immune-inflammation index with incident breast cancer and all-cause mortality: evidence from a large population-based study
Source: Front Immunol. 2025 Jan 24;16:1528690. doi: 10.3389/fimmu.2025.1528690 (PMC11802490; doi:10.3389/fimmu.2025.1528690)
Supplement: Supplementary file 1 [file Table1.docx]

Supplementary Table 1: Collinearity diagnostics steps.

|  | VIF |  |
| --- | --- | --- |
| SII | 1 | |
| Marital status | 1.1 | |
| Education | 1.3 | |
| Poverty-to-income ratio | 1.3 | |
| BMI | 1.1 | |
| Age | 1.2 | |
| Race | 1.1 | |
| Drinking | 1.1 | |
| Smoking | 1 | |
| Smoking | 1.1 | |

VIF: variance inflation factor; VIF = 1/(1-R^2^)

Abbreviations are as follows. SII: Systemic Immune Inflammation Index; BMI: Body Mass Index

Note: The variables with VIF>5 will be regarded as collinear variables and cannot be included in the multiple regression model.


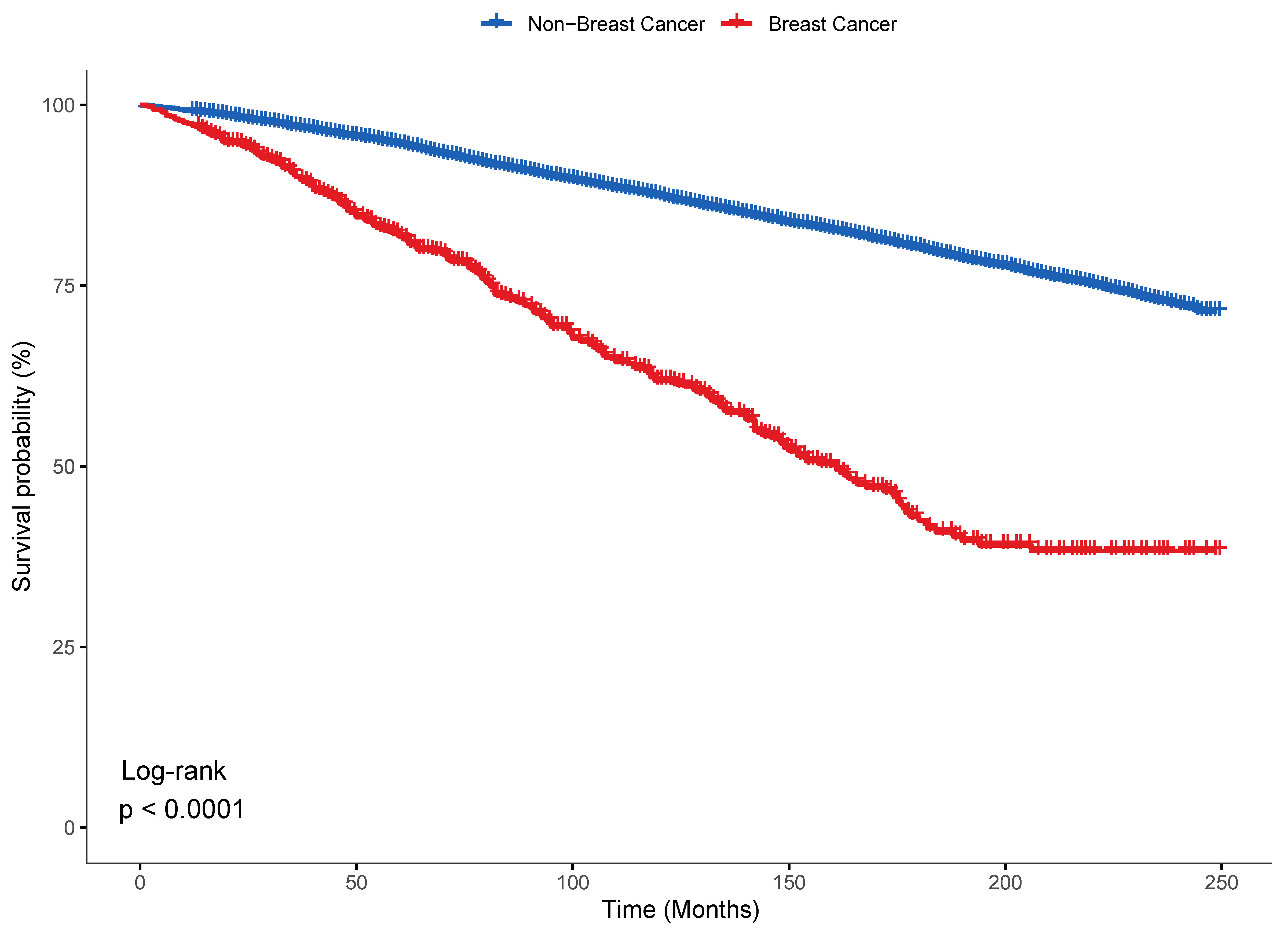


Supplementary Figure 1:
